# Multigenerational and Transgenerational Effects of Dioxins

**DOI:** 10.3390/ijms20122947

**Published:** 2019-06-17

**Authors:** Matti Viluksela, Raimo Pohjanvirta

**Affiliations:** 1 School of Pharmacy and Department of Environmental and Biological Sciences, University of Eastern Finland, P.O. Box 1627, FI-70211 Kuopio, Finland; 2 Environmental Health Unit, National Institute for Health and Welfare, P.O. Box 95, FI-70701 Kuopio, Finland; 3 Department of Food Hygiene and Environmental Health, Faculty of Veterinary Medicine, University of Helsinki, P.O. Box 66, FI-00014 Helsinki, Finland; raimo.pohjanvirta@helsinki.fi

**Keywords:** 2,3,7,8-tetrachlorodibenzo-*p*-dioxin (TCDD), dioxins, aryl hydrocarbon receptor, epigenetic modifications, transgenerational effects, gender ratio, preterm birth, paternal, maternal

## Abstract

Dioxins are ubiquitous and persistent environmental contaminants whose background levels are still reason for concern. There is mounting evidence from both epidemiological and experimental studies that paternal exposure to the most potent congener of dioxins, 2,3,7,8-tetrachlorodibenzo-*p*-dioxin (TCDD), can lower the male/female ratio of offspring. Moreover, in laboratory rodents and zebrafish, TCDD exposure of parent animals has been reported to result in reduced reproductive performance along with other adverse effects in subsequent generations, foremost through the paternal but also via the maternal germline. These impacts have been accompanied by epigenetic alterations in placenta and/or sperm cells, including changes in methylation patterns of imprinted genes. Here, we review recent key studies in this field with an attempt to provide an up-to-date picture of the present state of knowledge to the reader. These studies provide biological plausibility for the potential of dioxin exposure at a critical time-window to induce epigenetic alterations across multiple generations and the significance of aryl hydrocarbon receptor (AHR) in mediating these effects. Currently available data do not allow to accurately estimate the human health implications of these findings, although epidemiological evidence on lowered male/female ratio suggests that this effect may take place at realistic human exposure levels.

## 1. Introduction

It is a well-known fact that maternal lifestyle and exposure to environmental factors during pregnancy are important for the health of the offspring. According to the developmental origins of health and disease (DOHaD) concept, environmental exposures during critical windows of development may result in functional impairment, increased risk for diseases, and other long-term health consequences later in life [1,2]. Much less attention has been paid to the toxicological significance of parental preconceptional exposures, especially effects mediated via the paternal germline [3,4]. However, there is increasingly more experimental and epidemiological evidence for potentially adverse outcomes of paternal exposures, although the current health advisories fail to emphasize the role of future fathers for the health of their offspring. 

In addition to health effects of direct parental exposures on the offspring, more data are accumulating on health consequences of ancestral exposures in future generations. Understanding of the biological mechanism underlying non-genetic transgenerational inheritance of toxic effects have promoted the research of these phenomena. Paternal and transgenerational effects involve epigenetic mechanisms, and experimental studies have shown that environmentally induced epigenetic modifications of gametes may result in alterations in fertility, embryonic development, and health of the next generations [3,4,5].

As potent and persistent environmental contaminants, dioxins are still raising concerns on adverse effects on human health [6]. Due to intensive research, there are data available on the molecular mechanism of action of dioxins and their toxic effects both in experimental models and in humans. Essentially, dioxin-induced alterations have been shown be transferred to next generations predominantly via male germline after exposures during susceptibility windows of epigenetic reprogramming of primordial germ cells [7]. It is therefore worthwhile to include dioxins in further attempts to elucidate the characteristics and significance of epigenetically mediated transgenerational effects. In a recent minireview, Brehm and Flaws [8] summarized the transgenerational effects of a whole range of different types of endocrine disrupting chemicals on male and female reproduction. The purpose of this mini review is to focus on available data on paternally and maternally mediated multi- and transgenerational effects of dioxins. 

### 1.1. Epigenetic Alterations and Environmental Factors

**Figure 1:**
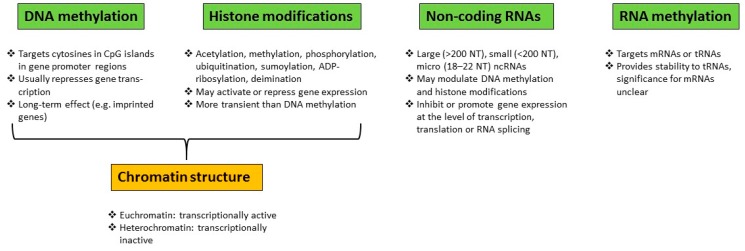
Epigenetic mechanisms affecting gene expression and their key features.

Epigenetics involves a variety of mechanisms that can regulate gene expression without alterations in the underlying DNA sequence in the genome. These include DNA methylation, histone modifications, non-coding RNAs, chromatin structure, and RNA methylations [5] (Figure 1). Epigenetic modifications play a significant role in normal development and they also allow organisms to adapt into a changing environment. However, altered gene expression and consequently altered phenotype may also result in toxic effects or disease states. From a toxicological point of view, an important property of epigenetic alterations in gametes is that when they occur in imprinted genes, they can be transmitted to subsequent generations. Imprinted genes are methylated in either the female (maternally imprinted) or the male (paternally imprinted) germline, and they retain this inheritance pattern in the next generation. In mammals, some 150 imprinted genes have been identified to date [9].

Previous studies have shown that exposure to different types of environmental and nutritional factors, such as chemicals, high-fat diet, caloric restriction, and stress during embryogenesis may result in adult-onset toxic effects or disease states for multiple generations [5,10,11,12]. These effects are called multigenerational when there is a direct exposure of the generation to the environmental factor, and in contrast, if the effects are transmitted in the germ line without continued involvement of direct exposure, they are transgenerational (also called ancestral exposure). If a pregnant female is exposed, there is a direct exposure of three generations: The female (F0 generation), the fetuses (F1 generation) and the germline of the fetuses (prospective F2 generation). Therefore, the F3 generation is the first unexposed generation, and effects observed in the F3 and subsequent generations are transgenerational. On the other hand, if a male or preconception female (F0) is exposed, also the germline (F1, eggs or sperm) is directly exposed, and in this case F2 generation is the first unexposed generation. Similarly, in fish and other species with external fertilization and embryonic development, exposure of parent animals (F0) involves also exposure of the F1 germline, and F2 generation is the first unexposed generation.

Upon fertilization, there is a rapid demethylation of most of the paternal (and slower for maternal) genome by the blastocyst stage, although e.g., imprinted genes show resistance. Soon after implantation, a wave of global de novo methylation occurs and is maintained in somatic cells. In primordial germ cells, however, another demethylation step (more substantial than the first one) follows, reaching its peak at embryonic days 11.5–12.5 in mice. In the male germline, de novo methylation is then soon initiated (at embryonic day 13.5 in mice), and the male methylome is completely established prior to birth. In contrast, de novo methylation does not begin until after birth in the female germline, reaching completion by postnatal day 21 in mice [13,14].

### 1.2. Dioxins

**Figure 2:**
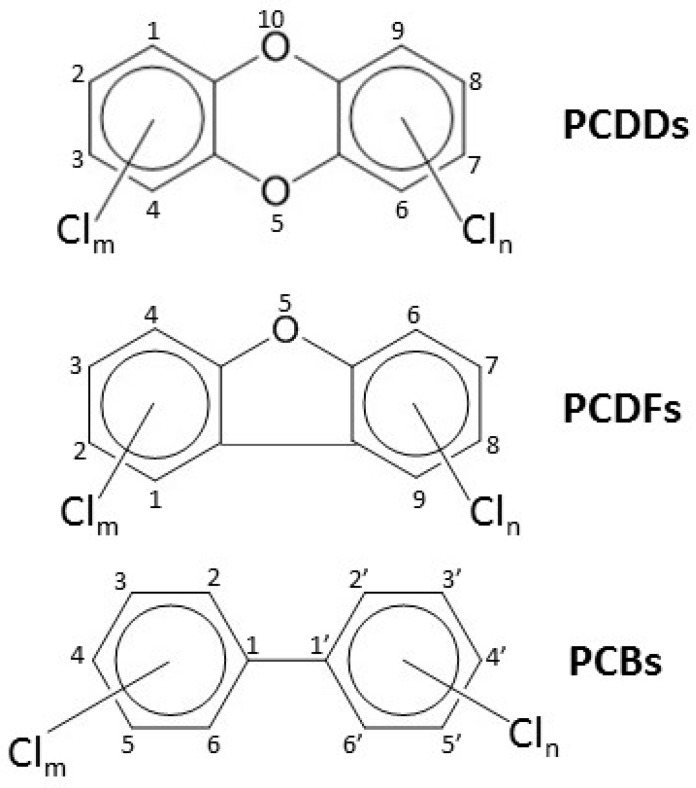
Structural formulas of dioxins. There are 75, 135, and 209 possible congeners of polychlorinated dibenzo-*p*-dioxins (PCDDs), polychlorinated dibenzofurans (PCDFs) and polychlorinated biphenyls (PCBs), respectively. For toxic PCDD/Fs (17 congeners), at least the lateral (*para*-) positions (2, 3, 7, 8) have chlorine substituents. For dioxin-like polychlorinated biphenyls (DL-PCBs) (12 congeners), there is maximally one chlorine atom in the *ortho*-positions (2, 2′, 6, 6′) and at least 4 chlorine atoms in the *meta*-(3, 5, 3′, 5′) and *para-*(4, 4′) positions.

“Dioxins” is the common name for a large number of potent and persistent environmental pollutants that include polychlorinated dibenzo-*p*-dioxins (PCDDs), polychlorinated dibenzofurans (PCDFs) and dioxin-like polychlorinated biphenyls (DL-PCBs) (Figure 2). PCDDs and PCDFs are formed as unwanted by-products in waste incineration at low temperatures and in industrial processes, but PCBs have been intentionally manufactured for a variety of industrial applications between 1929 and 1970s when they were banned. Due to the persistence and ability of dioxins to biomagnify in the food chain, humans are still exposed to them mainly via food. The most potent congener of dioxins is 2,3,7,8-tetrachlorodibenzo-*p*-dioxin (TCDD) which therefore serves as the toxicological prototype for the entire group. Dioxins are presumed to share a common mode of action and to display a purely additional co-effect (dose additivity). To facilitate the assessment of toxicity of dioxin mixtures, all dioxin congeners have been assigned a toxic equivalency factor (TEF) relative to TCDD (which has the TEF of 1). When the concentration of each congener in the mixture is multiplied by its TEF value and the resultant products are summed up, the result shows the amount of TCDD the mixture equals to in terms of its toxicity (TEQ).

In laboratory animals, dioxins bring about a wide variety of adaptive responses and toxic effects, ranging from induction of xenobiotic-metabolizing enzymes and endocrine imbalances to a peculiar wasting syndrome and carcinogenicity. The developing fetus is an exquisitely sensitive stage to their toxicity, and exposure to low doses of dioxins at this phase can result in both morphological and functional alterations that persist to, or only become visible at, adult age. In humans, the best established effect is chloracne, discovered already in the late 1950s [15]. However, it is a high-dose effect, usually requiring over 100-fold larger exposure than some of the most sensitive ones, which typically involve perinatal exposure, such as tooth mineralization defects and a low sperm number [6].

### 1.3. Aryl Hydrocarbon Receptor (AHR) and the Molecular Mechanism of Action of Dioxins

#### 1.3.1. Canonical Pathway

The AHR is a phylogenetically conserved protein that was found in mid-1970’s as an intracellular receptor for TCDD [16,17,18]. Induction of the cytochrome-P450 mono-oxygenase CYP1A1 by TCDD has subsequently been used as a model in studies elucidating the canonical pathway of AHR action. These and molecular cloning studies [19,20] have revealed that AHR is a ligand-activated transcription factor which functionally resembles steroid receptors, foremost glucocorticoid receptors, but structurally belongs to a fundamentally different protein superfamily, the bHLH/PAS (basic helix-loop-helix/Periodic-ARNT-Single-minded) proteins. Unliganded AHR resides in the cytosol in a protein complex also comprising a dimer of HSP90 and the co-chaperones AIP (also known as XAP2) and p23 [21]. The chaperones stabilize the AHR, maintain it in an optimal conformation for binding ligand, and inhibit its translocation into the nucleus [21,22,23,24]. Binding of TCDD to the AHR causes a transformation in its conformation [25,26], and the protein complex translocates into the nucleus [27]. There the AHR sheds its cytoplasmic partner proteins and dimerizes with a structurally related nuclear protein, AHR nuclear transporter (ARNT), to bind to the DNA at specific sites called dioxin response elements (DREs also known as xenobiotic-responsive elements (XREs) or AHR enhancers (AHREs)) in the promoter region of the *Cyp1a1* gene [28]. DRE binding is followed by changes in chromatin conformation, nucleosomal disruption over the transcribed region of the gene, and launching of mRNA transcription [29,30]. AHR activity is terminated by nuclear export of the receptor [31,32], and by its ubiquitin-mediated degradation by the 26S proteasome [32,33,34].

In addition to *Cyp1a1*, a number of other Phase I and Phase II biotransformation genes are consistently upregulated by TCDD-activated AHR, constituting the bulk of the so-called “AHR battery” of genes. In mammals, these include *Cyp1a2*, *Cyp1b1*, *Cyp2s1*, *Cyp2a5*, *Aldh3a1*, *Gsta1*, *Ugt1a6* (in humans, *UGT1A8*, *UGT1A9*, and *UGT1A10*) and *Nqo1* [35,36,37,38,39,40]. Besides them, TCDD alters the expression of hundreds of genes in adult mouse or rat liver, with little overlap across species [41,42,43]. However, the outcome can also be repression of gene activity. In fact, mere presence of functional AHR appears to mainly suppress gene activity in mice, whereas AHR activation by TCDD predominantly results in upregulation of gene expression [44]. The molecular mechanism(s) for transcriptional gene repression by the AHR are still poorly defined [45]. Moreover, among the genes induced by TCDD are at least two whose products act as suppressors of AHR activity, thus forming a feedback loop: AHR repressor (AHRR) and TCDD-inducible poly-ADPribose transferase (TiPARP) [46,47,48].

#### 1.3.2. Non-Canonical Pathway

Although a great majority of the biological effects of TCDD and other dioxins scrutinized to date have proven to be mediated through the canonical signaling route, the AHR has also other modes of action. In cultured cells, TCDD may elicit inflammatory changes by rapidly increasing Ca^2+^ concentration followed by activation of cytosolic phospholipase A2, c-SRC kinase, and cyclo-oxygenase 2. These effects require the AHR but not ARNT [49,50]. Alternatively, DRE binding is not involved in the AHR-mediated repressed expression of acute-phase proteins such as serum amyloid A 3 (Saa3) in mouse hepatocytes [51], in induction by TCDD of several early markers of inflammation in mouse liver and matrix metalloproteinase-1 in human bronchial cells [52,53], or in AHR-mediated suppression of genes involved in cholesterol synthesis in mouse liver in vivo [54]. A further different non-genomic action mechanism for TCDD-activated AHR has proven to be its functioning as a nuclear E3 ubiquitin ligase. Thereby, it directs estrogen and androgen receptors as well as β-catenin to proteasomal degradation [55,56].

The AHR acts in concert with other signaling systems in the cell having abundant cross-talk with them. Such interactions have been shown to exist, for example, with mitogen-activated protein kinases (MAPKs) [57], cyclic AMP/protein kinase A/phosphodiesterase 2A (cAMP/PKA/PDE2A) [58], transforming growth factor β (TGF-β) [59], tumor necrosis factor-α (TNF-α) [60], retinoblastoma (pRb), and E2 factor (E2F) [61,62,63], p53 [64], protein-tyrosine kinases and epidermal growth factor receptor (EGFR) [65,66,67], cyclins and cyclin-dependent kinases [68], nuclear factor erythroid 2-related factor 2 (Nrf2) [37,40], nuclear factor-κB (NF-κB) [69], and the Wnt/β-catenin signaling pathway [70].

Functional interactions of the AHR have also been demonstrated with a wide variety of other nuclear receptors including glucocorticoid, androgen, progesterone, constitutively active (CAR), pregnane X (PXR), thyroid hormone, liver X (LXR), retinoid acid/retinoid X (RAR/RXR), hypoxia-inducible factor-1α (HIF-1α), and peroxisome proliferator-activated receptors (PPARs) [71,72,73], reviewed in [74]. The most extensive cross-talk has been reported to occur with estrogen receptors (ER), including transcriptional repression of ER [75], disruption of the levels of the hormonal ligand [76], interference with ER signaling through inhibitory DREs [77], recruitment of ER to AHR-regulated genes thus diverting it from its own target genes [78], competition with ER for ARNT as a partner or coactivator [79], and targeting of ER to proteasomal destruction as described above. However, they may depend on cell type, promoter context, estrogen level, and receptor expression patterns [80].

##### Epigenetic Modifications

There is accumulating evidence of epigenetic modifications related to AHR signaling and TCDD toxicity [81]. For example, the AHR itself is a target of epigenetic regulation, as histone acetylation appears to be critical for the transcriptional activation of the *Ahr* promoter [82,83]. Epigenetic mechanisms also largely determine the induction ratios of CYP1A1 and CYP1B1 in various human and mouse cell lines upon TCDD treatment [84,85,86], and histone H3 phosphorylation at serine-10 in the DRE of *Cyp1a1* was demonstrated to be a prerequisite for the induction of *Cyp1a1, Aldh3a1*, and *Nqo1* in TCDD-treated mouse hepatoma cells, presumably via chromatin remodeling [87].

In nuclear extracts from Hepa 1c1c7 mouse hepatoma cells, methylation of *Cyp1a1* enhancer inhibited AHR binding to this site [88]. Exposure of preimplantation embryos to TCDD tended to decrease the expression levels of the imprinted genes *H19* and *Igf2*; this was associated with elevated methylation level of the *H19/Igf2* imprint control region [89]. In utero exposure to TCDD on gestation day (GD) 10 or 10.5 augmented global DNA methylation, histone acetyltransferase activity, and acetylated H3 level compared with the control 3 days later in palatal tissue of fetal mice, possibly being causally related to TCDD-induced cleft palate [90,91]. DNA methylation seems to play a role in other effects of TCDD too. TCDD enhanced promoter methylation of the tumor suppressors *p16(INK4a)* and *p53*, thereby repressing their transcription. This inhibited the senescence of primary human keratinocytes and immortalized them [92], a finding which may bear on dioxin carcinogenicity. TCDD also attenuated experimental colitis in mice by affecting the methylation status of CpG islands of *Foxp3* and *IL-17* promoters in T cells, thereby influencing reciprocal differentiation of Tregs and Th17 cells [93]. 

In addition to histone modifications and DNA methylation changes, TCDD may modulate the expression of non-coding RNAs. TCDD treatment of pregnant mice on GD 10 diminished expression of the lncRNA H19 in fetal palate on GD 13.5 but substantially augmented it a day later in comparison with the control. Concurrently, the expression pattern of a gene from the same imprinted locus, *Igf2,* displayed a mirror image [94]. Regarding micro-RNAs, although TCDD treatment of adult rodents affected the hepatic expression levels of only few micro-RNAs, prenatal exposure of mice to TCDD altered the expression levels of 78 micro-RNAs in fetal thymocytes by more than 1.5-fold [95,96]. Moreover, micro-RNA induction appears to be critical for attenuation of experimental autoimmune encephalomyelitis by TCDD [97]. 

Hence, TCDD (and most likely other dioxins), acting through the AHR, is capable of bringing about a wide variety of epigenetic changes in vivo and in vitro. When these occur in gametes, they have the potential to adversely affect the next and even subsequent future generations. Although the environmental emissions of dioxins, and consequently their concentrations in humans and biota, have declined markedly since 1970s, their current levels in foodstuffs still result in exceedance of their tolerable weekly intake level in the whole of Europe [6]. Especially sensitive targets to dioxins have proven to be the developing fetus and the reproductive system [6]. Therefore, we review here the data available at present on multi- and transgenerational effects of dioxins, focusing mainly on developmental and reproductive impacts and using TCDD as a prototype of dioxins.

## 2. Paternally or Maternally Mediated Effects on the Next Generation 

Toxicological consequences of paternal (in contrast to maternal) exposures to offspring are still poorly characterized, and there are no comprehensive studies addressing the whole spectrum of potential adverse effects. With regard to dioxins, findings of the currently available observational human studies are limited to lower male/female sex ratio of offspring observed in the next generation. In both humans and experimental animals, this effect appears to require paternal exposure. However, in mice also reduced fertility, preterm birth, and skeletal effects have been reported after either paternal or maternal exposure to dioxins. Human and experimental studies focusing on paternally versus maternally mediated effects of dioxins on the next generation offspring are summarized in Table 1.

### 2.1. Effects on Male/Female Sex Ratio

#### 2.1.1. Humans

The male/female sex ratio at birth has been shown to be relatively stable in different human populations, about 0.515 (proportion of males about 51.5% of total births) [107]. The sex ratio has therefore been proposed to be suitable to be used as a simple and non-invasive parameter for monitoring reproductive health of human populations [108]. Findings on male/female sex ratio in dioxin exposed populations have raised also general interest in paternally mediated effects. The observation of Mocarelli et al. [98] on lowered male/female sex ratio in the offspring of fathers exposed to TCDD during the Seveso accident in 1976 was the first to emphasize the toxicological significance of paternally mediated effects. The sex ratio [100 × boys/(boys + girls)] was lower (43.6%) if only the father was exposed and if both father and mother were exposed (serum TCDD concentration >15 pg/g lipid), but if only the mother was exposed, the sex ratio of offspring did not differ from the expected (Table 1A). The lowest sex ratio was observed in the offspring of fathers who were exposed at the age of less than 19 years (38.2%) even though the time of conception had been more than 15 years after the exposure. It seems therefore that the time before and during puberty is very sensitive to this effect and that the effect persists several years in spite of decreasing TCDD concentrations.

A similar decrease in male/female sex ratio was also observed among children of Russian pesticide workers who had been exposed to dioxins (mainly TCDD) during manufacture of trichlorophenol and 2,4,5-trichlorophenoxy acetic acid (2,4,5-T) [99]. Serum dioxin concentrations analyzed several years after occupational exposure were very similar in fathers and mothers and indicated median concentrations of 177 pg TEQ/g lipid (range 17–1930) for the 2,4,5-T cohort and 672 pg TEQ/g lipid (range 87–8520) for the trichlorophenol cohort. In both cohorts, the male/female sex ratio was decreased if father alone was exposed to dioxins (sex ratios 40 and 35%, respectively) or if both father and mother were exposed (sex ratios 41 and 39%, respectively). However, only maternal exposure resulted in the expected sex ratio of the offspring.

More recently, lower male/female sex ratio was reported in phenoxy herbicide workers exposed to TCDD in New Zealand [100]. In accordance with the earlier findings, lower sex ratio was associated with paternal (47%), but not maternal exposure to TCDD, and the probability of a male birth decreased with higher paternal serum TCDD concentration. Lower sex ratio was observed at exposure levels of ≥20 pg TCDD/g lipid.

In a systematic review published in 2011 addressing the influence of environmental and occupational hazards on the sex ratio at birth, Terrell et al. [108] analyzed the available studies on dioxin and PCB exposed populations. They concluded that paternal dioxin exposure is associated with a decreased proportion of male births, but PCB exposure (including both non-dioxin-like and dioxin-like PCBs) with a higher proportion of male births. However, in a more recent systematic review Nieminen et al. [109] did not find evidence that parental PCB exposure would alter the sex ratio of the offspring.

#### 2.1.2. Laboratory Animals

In earlier experimental studies in which both parents were exposed, the sex ratio of the offspring was not reported. In 2006, Rowlands et al. [110] re-examined the original data of the three-generation reproduction toxicity study in rats published by Murray et al. in 1979 [101]. They did not find altered sex ratio in any of the three generations of rats exposed continuously to TCDD (Table 2A). Contrary to these findings, in all later studies paternal only or both paternal and maternal exposure to TCDD was shown to lead to reduced male/female sex ratio, whereas maternal only exposure did not alter the sex ratio (Table 1B).

Significantly reduced male/female sex ratio (38%) was reported in the offspring (F2 generation) of male rats exposed to TCDD in utero and lactationally (F1 generation) and mated with unexposed females [102]. Maternal TCDD exposure from 2 weeks prior to mating until weaning (total dose ~1050 ng/kg) did not affect F1 generation mortality, litter size, sex ratio, or sperm numbers, but body weight at weaning and the ventral prostate weight were decreased. In F2 generation of paternal germline, no other effects except decreased sex ratio were reported (maternal germline was not studied). 

In another study, male mice were exposed to TCDD for 5 weeks using a loading dose/maintenance dose regimen (total doses 0, 4, or 4000 ng/kg bw) prior to mating with unexposed females [103]. Male/female ratio of the offspring (F1 generation) was dose-dependently decreased (48.8 and 46.2%, respectively). With the exception of increased liver weight in some high dose animals, no effects on body or organ weights, testicular histopathology, or epididymal sperm numbers were observed. Further studies from the same laboratory showed that at the higher dose-level, the epididymal sperm concentration, sperm motility, and the ratio of Y-bearing/X-bearing epididymal sperm were only slightly but nonsignificantly decreased [104]. However, the sex ratio of the 2-cell embryos was significantly decreased (Ctr: 53.95%, TCDD 47.92%), but the litter size was not affected. The magnitude of decrease was very similar with that observed at birth in the previous study at the same dose-level and without altered litter size. This suggests that the sex ratio of the offspring is decreased at fertilization resulting in a decreased sex ratio at birth, and that this decrease is mediated by reduced fertility of Y-bearing sperm.

When epididymal mouse sperm was exposed to TCDD for 1 h in vitro, sperm motility and viability were decreased concentration-dependently at 25 and 2500 ng/mL [106]. Interestingly, compared to the X-spermatozoa, the life-span of Y-spermatozoa was also decreased at the same concentrations. In an in vitro fertilization experiment, fertilization and embryonic development were decreased at the same concentrations. Although the proportion Y-spermatozoa was not affected at the lowest TCDD concentration of 0.25 ng/mL, the sex ratio of 2-cell embryos was significantly decreased already at this concentration and the decrease was concentration dependent. Based on these studies, it can be concluded that TCDD treatment of sperm in vitro results in decreased viability of Y-spermatozoa leading to decreased proportion of Y-spermatozoa. In addition, Y-spermatozoa have further a lower capability to fertilize oocytes than X-spermatozoa, and these changes are likely to contribute to the decreased male/female sex ratio after paternal dioxin exposure.

In addition to mammals, the effect of TCDD on sex ratio of offspring has also been studied in zebrafish (Table 2B). Baker et al. [119] exposed immature juvenile zebrafish to 50 pg/mL TCDD in water for 1 h at the age of 3 and 7 weeks post fertilization (F0 generation, both males and females were exposed). Male/female ratio was decreased in all studied generations (F0, F1 and F2), but the effect attenuated with time: F0 55.5%, F1 59.3% and F2 61.4% (normal male/female ratio in zebrafish is 67%). 

### 2.2. Effects on Pregnancy Outcome

Toxicological significance of paternal and maternal exposure on pregnancy outcomes was compared in mice exposed in utero and lactationally to TCDD [105]. Pregnant female mice (F0 generation) were given a single oral dose of 10 µg TCDD /kg bw on GD 15.5 (organogenesis completed) and the in utero and lactationally exposed F1 generation animals were mated with untreated mice of the opposite sex. The length of pregnancy of the F0 generation was not affected by TCDD treatment. In utero and lactationally exposed F1 males had reduced fertility (only 47% of their partners became pregnant) and, interestingly, gestational length of their unexposed partners was reduced (Ctr 20 days, TCDD 18.5 days). Also placental weights and pup weights were decreased and the mRNA expression of progesterone receptor (PGR) A and B was decreased and that of toll-like receptor 4 (TLR4) increased. Quite similarly, TCDD exposed F1 generation females mated with unexposed males had also reduced fertility (39% pregnant), preterm births (gestational length 18.5 days), reduced pup weight (but only nonsignificantly decreased placental weight), and the mRNA expression of PGR A and B decreased and that of TLR4 increased. None of the TCDD exposed F1 females mated with TCDD exposed F1 males became pregnant. At the end of pregnancy, decreased progesterone action promotes parturition and has been linked to preterm birth both in humans and in mice [121]. TCDD-induced preterm births are the likely consequence of decreased placental expression of PGRs together with TLR4 mediated increase in inflammatory cytokine production. The outcome of this study emphasizes the significant role of preconception paternal exposure in pregnancy outcomes of unexposed partners. This is in accordance with the fact that paternally derived genes play a significant role in placental development [122].

## 3. Paternally or Maternally Mediated Multigenerational and Transgenerational Effects

Experimental studies reporting paternally or maternally mediated effects on multiple generations (F1–F3) are summarized in Table 2. In a subsequent study from the same laboratory, Bruner-Tran and Osteen [114] also reported decreased fertility and increased incidence of preterm births in in utero and lactationally exposed F1 female mice mated with unexposed males. Interestingly, without further TCDD exposure reduced fertility (44, 43, and 55% pregnant in F1, F2, and F3 generations, respectively) and preterm births (and consequently increased neonatal mortality) were also observed in F2 and F3 generations (gestation length typically 17–18 days in F1, F2, and F3 generations; 20 days in controls). In accordance with earlier findings, immunostaining of PGR A and B isoforms was greatly reduced in uteri of infertile F1 and F4 females. For comparison, paternally mediated transgenerational effects on pregnancy outcome were demonstrated in a further study in which TCDD lineage F1 and F2 male mice were mated with unexposed females and the pregnancy outcome monitored in F1–F3 generations [115]. Fertility was decreased in F1–F3 generations (47, 48, and 50% pregnant in F1, F2, and F3 generations, respectively). Sperm morphology was within normal limits in all generations, but sperm concentration was decreased by about 35% in F1 generation. Preterm births were increased in all generations (average gestation length 18.6, 18.8, and 19 days in F1, F2, and F3 generations, respectively; 20 days in controls). AHR expression in spermatocytes was increased in all three generations. Accordingly, its increased expression has been associated with infertility in humans [123] and with toxic exposures in mice [124]. In addition, males of all three generations exhibited a hyper-inflammatory testicular phenotype characterized by loss of prostaglandin dehydrogenase, a trend for increased testicular prostaglandin E_2_, increased apoptosis of developing spermatocytes, and increased number of resident macrophages. There was also a trend towards decreased serum free testosterone levels. 

The changes observed in the reproductive system of F3 males strongly suggest that epigenetic modifications are mediating the effects observed within paternal germline. In order to study further the effects on sperm and placental epigenome, a global methylation focused microarray analysis was carried out on the paternal germline F1 and F3 generations [116]. The analysis identified altogether 2171 differentially methylated regions. In F1 and F3 generation placentae, 15% of genes were differentially methylated, and the promoter region of *Pgr* was hypermethylated and that of insulin-like growth factor-2 (*Igf2*) hypomethylated. The mRNA expression of *Pgr* and *Igf2* was decreased, and expression of IGF2 protein was also decreased. Expression of PGR protein was decreased in junctional and labyrinth zones of placentae. *Igf2* is a paternally expressed imprinted gene that promotes fetal growth. Hypomethylation of *Igf2* is usually linked with decreased mRNA expression of this gene. It is noteworthy that both placental weight and pup weight were decreased in both F1 and F3 generations demonstrating intrauterine growth restriction. This study showed that in utero and lactational dioxin exposure of male mice may modify placental epigenome resulting in placental dysfunction and adverse pregnancy outcomes in unexposed mating partners. Importantly, these adverse outcomes may be inherited transgenerationally to unexposed generations. Since gestation length was shortened even more if females were exposed, placental dysfunction plausibly contributes to the transgenerational preterm births recorded after either ancestor or ancestress exposure in mice.

Effects of a single oral dose of 0.1, 0.5, or 1.0 µg TCDD/kg bw given to pregnant rats on GD 15 were studied in the paternal germline offspring in F1–F3 generations by Sanabria et al. [117]. F1 generation males exposed in utero and lactationally to TCDD were mated with unexposed females to obtain the F2 and further the F3 generation. Serum testosterone levels and the transit time of sperm through the epididymis were dose-dependently decreased in F1 males (significant only at 1.0 µg/kg bw; for sperm transit time *p* = 0.07). Also, the proportion of morphologically normal sperm was decreased dose-dependently in F1 males at 0.5 and 1.0 µg/kg bw. The most significant finding manifest in all three generations was a decreased proportion of implantations per corpus luteum (significant decreases: F1 at 0.5 and 1.0 µg/kg bw, F2 at 0.1 µg/kg bw, F3 at 0.1, 0.5, and 1.0 µg/kg bw; Table 2B). 

Effects of paternal and maternal TCDD exposure on egg release and egg fertilization in F1 and F2 zebrafish generations were compared in the above mentioned (see Section 2.1.2) study by Baker et al. [119]. When TCDD lineage F1 males were mated with unexposed females, the elicitation of egg release and egg fertilization success were decreased both in F1 and, slightly less, in F2 generation. When TCDD lineage F1 females were mated with untreated males, egg release was also decreased, but less than after paternal exposure, and the decrease did not achieve statistical significance in F2 generation. Maternal exposure did not affect the egg fertilization. Based on these findings, alterations in TCDD lineage males are largely responsible for decreased egg release and fertilization. In a subsequent study from the same laboratory, Meyer et al. [120] identified numerous differentially methylated genes in the testis of males of all three generations. In seminiferous tubules of F1 males, the number of spermatogonia was increased, but the number of spermatozoa decreased. In addition, Baker et al. [119] reported that exposure of F0 generation resulted in atretic ovarian follicles in females of F0, F1, and F2 generation with decreasing frequency (nonsignificant in F2 generation). Similarly, skeletal abnormalities including axial kinks, cranial malformations, and jaw malformations, were also observed in all three generations with decreasing frequency. This study showed that TCDD induces paternally mediated and transgenerational effects that include lowered male/female sex ratio (see Section 2.1), reproductive dysfunction, reduced fertility, and skeletal malformations. These effects seem to be phenotypically at least partly similar in fish and mammals. 

## 4. Paternally and Maternally Mediated Multigenerational and Transgenerational Effects

Studies of Manikkam et al. [111] described TCDD-induced epigenetically inherited transgenerational adult-onset adverse effects together with differentially methylated regions in sperm DNA. Pregnant female rats were exposed to 100 ng TCDD/kg bw/day i.p. (total dose 700 ng/kg bw) on GDs 8–14 covering the erasure and de novo methylation of male primordial germ cells. In subsequent generations (F1 and F2), animals were mated with the opposite sex of the same treatment group (control or TCDD). Studies on F1 and F3 generations showed delayed onset of puberty in males, pubertal anomalies in F1 females and early onset of puberty in F3 females. Prostate and kidney weights were decreased in F1 males and kidney changes characterized by fluid filled cysts, decreased glomerular size, and thickening of Bowman’s capsule in F3 males. In addition, F3 males had atrophic prostate duct epithelium as a sign of prostate disease. Sperm number or motility were not affected by the treatment. The occurrence of testis abnormalities did also not differ in a statistically significant manner in F1 or F3 generations. In females, polycystic ovary disease was more frequent in the F1 and F3 progeny of dioxin-treated F0 rats than in their control counterparts. Moreover, total disease incidence was elevated in the TCDD-lineage in both F1 and F3 generations in females but only in F1 males. An additional analysis of F3 generation sperm epigenome revealed 50 differentially methylated regions between TCDD and control lineages, but these were not predominant in specific cellular pathways [112]. Thus, exposure of rat dams to a relatively high dosage of TCDD during a critical time-window of development may bring about adverse transgenerational adult-onset health effects in both female and male descendants. These are accompanied by sperm epimutations, but the nature of the relationship is not clear.

TCDD-induced epigenetic transgenerational ovarian disease was further studied by Nilsson et al. [113]. The phenotype of polycystic ovary disease observed in F1 and F3 females was characterized by an increased number of small and large ovarian cysts (statistically significant only for small cysts in F3 females). Furthermore, the number of primordial follicles was significantly reduced in both F1 and F3 generation in the TCDD lineage, whereas developing preantral or antral follicles were not affected. Thus, the resting pool of oocytes was transgenerationally diminished and the normal follicle development disturbed by TCDD exposure.

## 5. Conclusions and Future Prospects

In humans, rodents and zebrafish, paternal (or paternal and maternal) exposure to TCDD has been reported to result in a lowered male/female ratio in offspring. Additional in vitro studies on mouse sperm suggest that this could be at least partly due to TCDD-induced reduction in survival and fertilization capability of spermatozoa carrying the Y chromosome. Male mice exposed perinatally to TCDD and mated with unexposed females have diminished fertility and the gestational length of their partners is shortened, possibly because of lowered PGR but elevated TLR4 abundance (shown for their mRNAs) in placenta. Similarly, in utero and lactational exposure to TCDD of either male or female F1 mice can reduce fertility and the length of gestation at least up to F3 generation. These phenomena are associated with a decreased abundance of PGR isoforms A and B in uterus in the case of maternal germline and increased AHR expression in spermatocytes for paternal germline. In the latter case, a hyper-inflammatory testicular phenotype has also been recorded. Moreover, paternally mediated effects have been found to be accompanied by a large number of differentially methylated regions in the DNA of sperm and placenta.

In rats, paternal germline has exhibited a decreased proportion of implantations per corpus luteum after TCDD exposure. In an experimental setting where descendants of dioxin exposed F0 rat dams are mated with one another, a number of abnormalities have been noticed in the F3 generation, including elevated total disease incidence and a reduced number of ovarian primordial follicles.

In zebrafish, egg release and egg fertilization have proven to be transgenerationally impaired after TCDD exposure. These studies also reported skeletal effects of TCDD up to the F2 generation.

Most of the studies assessing the mode of inheritance of TCDD-induced transgenerational impacts have so far assessed paternal germline effects alone. In those cases where these have been contrasted with maternal effects (in mice and zebrafish), the outcomes have proven to be qualitatively similar. However, at least in zebrafish, the magnitude of the effects was larger in the case of paternal germline. 

The available data provide the proof of principle on the potential of dioxins to induce epigenetic alterations and related toxic effects across multiple generations. On the other hand, the significance of these findings for human health is difficult to assess as there is too little information at present on the dose-responses of the multi- and transgenerational effects of TCDD. At present, it appears that these phenomena require fairly high exposures (close to the doses causing teratogenic manifestations). The available rodent studies have been carried out at dose levels between 0.1 and 10 µg/kg bw that are associated with dioxin body burdens clearly above the current human background exposure levels [6,125]. The most sensitive effect reported so far was the decreased proportion of implantations per corpus luteum in the rat offspring of F2 and F3 generations at 0.1 µg/kg bw [117]. However, it is important to note that the human studies on lowered male/female ratio in the offspring of TCDD exposed fathers suggest that this effect may take place at relatively low exposure level, starting at serum TCDD concentrations >15 pg/g fat [98] or ≥20 pg/g fat [100]. 

Transgenerational impacts of TCDD have emerged in conjunction with cellular epigenetic modifications, including altered methylation patterns of imprinted genes. Verifying whether this is causal relationship and pinpointing the key genes thus affected require further studies in the future. Likewise, the indispensable role of the AHR in these phenomena awaits formal verification using AHR-deficient animals. The molecular mechanisms upon AHR binding that lead to epigenetic alterations and the capability of other AHR agonists to cause them and transgenerational health effects will be further important research topics. Finally, with regard to the DoHaD concept the human health significance of the dioxin-induced preconceptional paternally mediated and transgenerational effects should be addressed in future studies.

## Figures and Tables

**Table 1:** Summary of human and experimental studies with a focus on paternally (versus maternally) mediated effects of dioxins on the next generation offspring. Reported changes statistically significant unless indicated nonsignificant (ns).

**A. Human Studies**						
**Population**	**Dioxin Exposure**		**Effects Mediated via Paternal or Maternal Germline**			**Reference**
			**Paternal Germline**	**Maternal Germline**	**Maternal and Paternal Germline**	
Seveso population	Serum TCDD concentrations Unexposed: ≤15 pg/g fat Exposed: >15 pg/g fat Fathers: median 96.5, range 2.8–26,400 pg/g fat Mothers: median 62.8, range 6.45–12,500 pg/g fat		Male/female ratio ↓: Unexposed 55.7%, exposed 43.6% Fathers <19 years at exposure: Male/female ratio ↓: Unexposed 53.5%, exposed 38.2%	Male/female ratio not changed: 54.5% (ns)	Male/female ratio ↓: 44.2%	Mocarelli et al., 2000 [98]
Russian pesticide producers	Serum TEQ concentration (mainly TCDD): Unexposed: not reported Exposed: median 243 pg/g fat, range 17–8520 pg/g fat		Male/female ratio ↓: Unexposed 51%, exposed 38%; higher exposed cohort with median 715 pg/g fat: 23%	Male/female ratio not changed (51%, ns)	Male/female ratio ↓: Unexposed 51%, exposed 40%	Ryan et al., 2002 [99]
New-Zealand phenoxy herbicide producers	Serum TCDD concentration back-calculated to time of offspring’s birth (4 categories): <4, 4–20, 20–100 and ≥100 pg/g fat		Male/female ratio ↓: TCDD <20 pg/g fat: 60% TCDD ≥20 pg/g fat: 47%	Male/female ratio not changed: TCDD <20 pg/g fat: 53% TCDD ≥20 pg/g fat: 68%, ns	No data	Mannetje et al., 2017 [100]
**B. Experimental Studies**						
**Species, Strain**	**Dosing of TCDD**		**Effects Mediated via Paternal or Maternal Germline**			**Reference**
	**Dose**	**Timing**	**Paternal Germline**	**Maternal Germline**	**Maternal and Paternal Germline**	
Rat, Sprague Dawley	0.1 µg/kg bw/day, in diet	12 months starting 90 days prior to mating, TCDD exposed F0 males and females mated with unexposed partners	Cross-mating study: No harmful effects on pregnancy or resorptions.	Cross-mating study: No harmful effects on pregnancy, resorptions ↑	Cross-mating study: Not examined	Murray et al., 1979 [101]
Rat, Holzman	Loading dose 400 ng/kg bw + maintenance doses 80 ng/kg bw/week Adipose tissue TCDD conc.: F0 dams GD 20: 1810, weaning: 840; F1 pups PND 28: ~480 pg/g wet weight	F0 females exposed 2 weeks before mating until end of lactation. TCDD exposed F1 males mated with unexposed females	F2: Male/female ratio ↓ (Ctr 52.2%, TCDD 38%).	Not examined	Not examined	Ikeda et al., 2005 [102]
Mouse, ICR	Loading dose 2 ng/kg bw + maintenance doses 5 × 0.4 ng/kg/bw/week or 2000 ng/kg bw + 5 × 400 ng/kg bw/week, oral gavage in sesame oil	5 weeks before mating, TCDD exposed males mated with unexposed females	F1: Male/female ratio ↓: Ctr 53.1%, TCDD 2/0.4: 48.8% (ns), TCDD 2000/400: 46.2%	Not examined	Not examined	Ishihara et al., 2007 [103]
Mouse, ICR	Loading dose 2000 ng/kg bw + maintenance doses 5 × 400 ng/kg bw/week, oral gavage in sesame oil	5 weeks before mating, TCDD exposed males mated with nonexposed females	Y-bearing/X-bearing sperm ratio ↓ (ns, Ctr: 2.68, TCDD: 2.36), sperm Sry DNA concentration ↓ (ns, Ctr 28.12, TCDD 25.80), male/female ratio of 2-cell embryos ↓ (Ctr: 53.95%, TCDD 47.92%)	Not examined	Not examined	Ishihara et al., 2010 [104]
Mouse, C57Bl/6	10 µg/kg bw, single dose, oral gavage in corn oil	GD 15.5 TCDD exposed F1 males mated with unexposed females and TCDD exposed F1 females mated with unexposed males	F: fertility ↓ (47% pregnant), premature births ↑ (Ctr 20 days, TCDD 18.5 days); placental weight ↓, pup weight ↓, placental progesterone receptor A and B ↓ and toll-like receptor-4 mRNA expression ↑, sensitivity to inflammation ↑	F: fertility ↓ (39% pregnant); premature births ↑, pup weight ↓, placental progesterone receptor A and B ↓ and toll-like receptor-4 mRNA expression ↑, sensitivity to inflammation ↑	F: fertility ↓ (0% pregnant)	Ding et al., 2011 [105]
Mouse, ICR	Epididymal sperm exposed to 0, 0.25, 25, or 2500 ng/mL in vitro	Incubation time 1 h	Sperm motility and viability concentration dependently ↓, acrosome-reacted spermatozoa ↑ at 25 and 2500 ng/mL, Y-spermatozoa survival concentration dependently ↓ at 25 and 2500 ng/mL, fertilization and early embryonic development in vitro ↓ at 25 and 2500 ng/mL, male/female ratio of 2-cell embryos dose-dependently ↓ at 0.25, 25, and 2500 ng/mL, male/female ratio of blastocysts concentration dependently ↓ at 25 and 2500 ng/mL	Not examined	Not examined	You et al., 2018 [106]

**Table 2:** Summary of experimental studies on multigenerational and transgenerational (F1–F3) effects of 2,3,7,8-tetrachlorodibenzo-p-dioxin (TCDD) on male (M) and female (F) offspring. Reported changes statistically significant unless indicated nonsignificant (ns).

**A. Rodent studies: both paternal and maternal exposure**						
**Species, Strain**	**Exposure to TCDD**		**Effects**			**Reference**
	**Dose**	**Timing**	**F1 Generation**	**F2 Generation**	**F3 Generation**	
Rat, Sprague Dawley	0.001, 0.01 or 0.1 µg/kg bw/day, in diet	90 days prior to mating throughout 3 generations (continuous exposure)	0.001 µg/kg: slightly dilated renal pelvis ↑, 0.01 µg/kg: time from cohabitation to delivery ↑, fertility ↓, postnatal survival ↓, 0.1 µg/kg: fertility ↓, litter size ↓, gestation survival index ↓ (discontinued)	0.001 µg/kg: no effects, 0.01 µg/kg: body weight ↓, time from cohabitation to delivery ↑, fertility ↓, litter size ↓, gestation survival index ↓, postnatal survival ↓	0.001 µg/kg: no effects, 0.01 µg/kg: body weight ↓, litter size ↓, gestation survival index ↓	Murray et al., 1979 [101]
Rat, Sprague Dawley	0.001, 0.01, or 0.1 µg/kg bw/day, in diet	90 days prior to mating throughout 3 generations	Male/female ratio not changed	Male/female ratio not changed	Male/female ratio not changed	Rowlands et al., 2006 [110] (re-examination of the Murray et al. 1979 data 99])
Rat, Sprague Dawley	100 ng/kg/day ip in DMSO Total dose: 700 ng/kg/day	GD 8-14	M: delayed puberty onset; testis weight ↑, prostate and kidney weight ↓ F: pubertal abnormalities; body weight ↓, ovarian primordial follicles ↓, polycystic ovary disease	Not examined	M: delayed puberty onset; kidney: weight ↓, fluid filled cysts, glomerular size ↓, thickening of Bowman’s capsule; serum testosterone ↑, 50 differentially methylated regions in sperm DNA, atrophic prostate duct epithelium F: early onset of puberty, kidney weight ↓, ovarian primordial follicles ↓, polycystic ovary disease	Manikkam et al., 2012a,b [111,112]; Nilsson et al., 2012 [113]
Rat, Sprague Dawley	100 ng/kg/day ip in DMSO Total dose: 700 ng/kg/day	GD 8-14	F: ovarian primordial follicles ↓, polycystic ovary disease: small ovarian cysts ↑ (ns), large ovarian cysts ↑ (ns)	Not examined	F: ovarian primordial follicles ↓, polycystic ovary disease: small ovarian cysts ↑, large ovarian cysts ↑ (ns)	Nilsson et al., 2012 [113]
**B. Rodent studies: paternal or maternal exposure**						
Mouse, C57Bl/6	10 µg/kg, single dose, oral gavage in corn oil	GD 15.5 TCDD exposed F1 and F2 females mated with unexposed males	F: fertility ↓, premature births ↑, progesterone receptor immunostaining in uterus of infertile mice ↓, sensitivity to inflammation ↓	F: fertility ↓, premature births ↑	F: fertility ↓, premature births ↑ (in F4: progesterone receptor immunostaining in uterus of infertile mice ↓)	Bruner-Tran and Osteen, 2011 [114]
Mouse, C57Bl/6	10 µg/kg, single dose, oral gavage in corn oil	GD 15.5 TCDD exposed F1 and F2 males mated with unexposed females	M: fertility ↓ (47% pregnant), premature births in unexposed partners ↑, sperm concentration ↓, normal sperm morphology ↓, sperm AHR expression ↑, testicular inflammation and apoptosis ↑	M: fertility ↓ (48% pregnant), premature births in unexposed partners ↑, normal sperm morphology ↓, sperm AHR expression ↑, testicular inflammation and apoptosis ↑	M: fertility ↓ (50% pregnant), premature births in unexposed partners ↑, normal sperm morphology ↓, sperm AHR expression ↑, testicular inflammation and apoptosis ↑	Bruner-Tran et al., 2014 [115]
Mouse, C57Bl/6	10 µg/kg, single dose, oral gavage in corn oil	GD 15.5 TCDD exposed F1 and F2 males mated with unexposed females	Placental weight ↓, pup weight ↓ F: 15% of genes in placenta differentially methylated, hypermethylation of progesterone receptor (*Pgr*), hypomethylation of insulin-like growth factor-2 (*Igf2*, ns), mRNA of *Pgr-b*, *Pgr-a/b*, *Igf-2*, and *H19* ↓, IGR2 protein ↓, mRNA of DNA methyltransfereases ↑ *Dnmt1*, *Dnmt3a* (ns), *Dnmt3b* (ns) M: in sperm hypermethylation of *Pgr*, hypomethylation of *Igf2*	Not examined	Placental weight ↓, pup weight ↓ F: 15% of genes in placenta differentially methylated, hypermethylation of *Pgr*, hypomethylation of *Igf2* (ns). M: in sperm hypermethylation of *Pgr* (ns) and hypomethylation of *Igf2* (ns), mRNA of *Pgr-b*, *Pgr-a/b* (ns), *Igf-2* (ns) and *H19* ↓, IGR2 protein ↓, mRNA of DNA methyltransfereases ↑ *Dnmt1*, *Dnmt3a* (ns), *Dnmt3b* (ns)	Ding et al., 2018 [116]
Rat, Wistar	0.1, 0.5 or 1.0 µg/kg bw, single dose, oral gavage in corn oil	GD15 TCDD exposed F1 and F2 males mated with unexposed females	M: serum testosterone ↓ (dose-dependent, only 1.0 significant), sperm transit time ↓ (ns), normal sperm morphology ↓ (0.5 and 1.0) F: implants per corpora lutea ↓ in unexposed partners at 0.5 and 1.0 µg/kg bw: Ctr 75.2%, 0.5 62.0%, 1.0 58.7%	F: implants per corpora lutea ↓ in unexposed partners at 0.1, and 1.0 µg/kg bw: Ctr 61.9%, 0.1 41.1%, 0.5 50.5% (ns), 1.0 43.6%	F: implants per corpora lutea ↓ in unexposed partners at 0.1, 0.5 and 1.0 µg/kg bw: Ctr 82.4%, 0.1 50.7%, 0.5 56.6%, 1.0 31.8%	Sanabria et al., 2016 [117]
**C. Zebrafish studies**						
			**F0 generation**	**F1 generation**	**F2 generation**	
Zebrafish	20 µg/kg in diet	Parental exposure 47 days		No effect on global DNA methylation in liver, CYP 1A1 ↑	No effect on global DNA methylation in liver	Olsvik *et al.*, 2014 [118]
Zebrafish AB strain	50 pg/mL in water (dissolved in DMSO)	1 h at week 3 and week 7 post fertilization	Male/female ratio ↓ (Ctr 71.1%, TCDD 55.5%) F: atretic ovarian follicles (65.5%), egg release ↓, fertilization success ↓ Skeletal abnormalities (82.4%) axial kinks (54.5%) cranial malformations (46.9%) jaw malformations (34.5%)	Male/female ratio ↓ (Ctr 70.8%, TCDD 59.3%) F: atretic ovarian follicles (46.1%), egg release ↓ M: elicitation of egg release ↓, fertilization success ↓ Skeletal abnormalities (34.9%) axial kinks (28.1%) cranial malformations (11.7%) jaw malformations (3.7%, ns)	Male/female ratio ↓ (Ctr 78.7%, TCDD 61.4%) F: atretic ovarian follicles (7.7%, ns) M: elicitation of egg release ↓, fertilization success ↓ Skeletal abnormalities (22.1%) axial kinks (17.3%) cranial malformations (7.8%, ns) jaw malformations (0.9%, ns)	Baker et al., 2014 [119]
Zebrafish AB strain	50 pg/mL in water (dissolved in DMSO)	1 h at week 3 and week 7 post fertilization	M: in testis 722 differentially expressed genes	M: in seminiferous tubules spermatogonia ↑, spermatozoa ↓, in testis 634 differentially expressed genes	M: in seminiferous tubules spermatozoa ↓ (ns), in testis 1105 differentially expressed genes	Meyer et al., 2018 [120]

## References

[1] Schug T.T., Barouki R., Gluckman P.D., Grandjean P., Hanson M., Heindel J.J. (2013). PPTOX III: Environmental stressors in the developmental origins of disease—Evidence and mechanisms. Toxicol. Sci..

[2] Hanson M.A., Gluckman P.D. (2014). Early developmental conditioning of later health and disease: Physiology or pathophysiology?. Physiol. Rev..

[3] Soubry A. (2018). Epigenetics as a Driver of Developmental Origins of Health and Disease: Did We Forget the Fathers?. Bioessays.

[4] Soubry A. (2018). POHaD: Why we should study future fathers. Environ. Epigenet..

[5] Nilsson E.E., Sadler-Riggleman I., Skinner M.K. (2018). Environmentally induced epigenetic transgenerational inheritance of disease. Environ. Epigenet..

[6] Knutsen H.K., Alexander J., Barregård L., Bignami M., Bruschweiler B., Ceccatelli S., Cottrill B., Dinovi M., Edler L., EFSA Panel on Contaminants in the Food Chain (CONTAM) (2018). Risk for animal and human health related to the presence of dioxins and dioxin-like PCBs in feed and food. EFSA J..

[7] Pilsner J.R., Parker M., Sergeyev O., Suvorov A. (2017). Spermatogenesis disruption by dioxins: Epigenetic reprograming and windows of susceptibility. Reprod. Toxicol..

[8] Brehm E., Flaws J.A. (2019). Transgenerational effects of endocrine disrupting chemicals on male and female reproduction. Endocrinology.

[9] Hanna C.W., Kelsey G. (2014). The specification of imprints in mammals. Heredity.

[10] Skinner M.K. (2008). What is an epigenetic transgenerational phenotype? F3 or F2. Reprod. Toxicol..

[11] Skinner M.K. (2014). Endocrine disruptor induction of epigenetic transgenerational inheritance of disease. Mol. Cell. Endocrinol..

[12] Marczylo E.L., Jacobs M.N., Gant T.W. (2016). Environmentally induced epigenetic toxicity: Potential public health concerns. Crit. Rev. Toxicol..

[13] Chen Z.X., Riggs A.D. (2011). DNA methylation and demethylation in mammals. J. Biol. Chem..

[14] Stewart K.R., Veselovska L., Kelsey G. (2016). Establishment and functions of DNA methylation in the germline. Epigenomics.

[15] Kimmig J., Schulz K.H. (1957). Chlorierte aromatische Zyklische Äther als Ursache der sogenannten Chlorakne. Natürwissenschaften.

[16] Niwa A., Kumaki K., Nebert D.W., Poland A.P. (1975). Genetic expression of aryl hydrocarbon hydroxylase activity in the mouse. Distinction between the "responsive" homozygote and heterozygote at the Ah locus. Arch. Biochem. Biophys..

[17] Atlas A.S., Thorgeirsson S.S., Boobis A.R., Kumaki K., Nebert D.W. (1975). Differential induction of murine Ah locus-associated monooxygenase activities in rabbit liver and kidney. Biochem. Pharmacol..

[18] Poland A., Glover E., Kende A.S. (1976). Stereospecific, high affinity binding of 2,3,7,8-tetrachlorodibenzo-p-dioxin by hepatic cytosol. Evidence that the binding species is receptor for induction of aryl hydrocarbon hydroxylase. J. Biol. Chem..

[19] Ema M., Sogawa K., Watanabe N., Chujoh Y., Matsushita N., Gotoh O., Funae Y., Fujii-Kuriyama Y. (1992). cDNA cloning and structure of mouse putative Ah receptor. Biochem. Biophys. Res. Commun..

[20] Burbach K.M., Poland A., Bradfield C.A. (1992). Cloning of the Ah-receptor cDNA reveals a distinctive ligand-activated transcription factor. Proc. Natl. Acad. Sci. USA..

[21] Murray I.A., Perdew G., Pohjanvirta R. (2012). Role of chaperone proteins in AHR function. The AH Receptor in Biology and Toxicology.

[22] Kazlauskas A., Poellinger L., Pongratz I. (1999). Evidence that the co-chaperone p23 regulates ligand responsiveness of the dioxin (Aryl hydrocarbon) receptor. J. Biol. Chem..

[23] Kazlauskas A., Poellinger L., Pongratz I. (2000). The immunophilin-like protein XAP2 regulates ubiquitination and subcellular localization of the dioxin receptor. J. Biol. Chem..

[24] Kazlauskas A., Sundstrom S., Poellinger L., Pongratz I. (2001). The hsp90 chaperone complex regulates intracellular localization of the dioxin receptor. Mol. Cell. Biol..

[25] Henry E.C., Gasiewicz T.A. (2003). Agonist but not antagonist ligands induce conformational change in the mouse aryl hydrocarbon receptor as detected by partial proteolysis. Mol. Pharmacol..

[26] Soshilov A., Denison M.S. (2008). Role of the Per/Arnt/Sim domains in ligand-dependent transformation of the aryl hydrocarbon receptor. J. Biol. Chem..

[27] Richter C.A., Tillitt D.E., Hannink M. (2001). Regulation of subcellular localization of the aryl hydrocarbon receptor (AhR). Arch. Biochem. Biophys..

[28] Swanson H., Pohjanvirta R. (2012). Dioxin response elements and regulation of gene transcription. AH Receptor in Biology and Toxicology.

[29] Okino S.T., Whitlock J.P. (1995). Dioxin induces localized, graded changes in chromatin structure: Implications for Cyp1A1 gene transcription. Mol. Cell. Biol..

[30] Hankinson O. (2005). Role of coactivators in transcriptional activation by the aryl hydrocarbon receptor. Arch. Biochem. Biophys..

[31] Ikuta T., Eguchi H., Tachibana T., Yoneda Y., Kawajiri K. (1998). Nuclear localization and export signals of the human aryl hydrocarbon receptor. J. Biol. Chem..

[32] Davarinos N.A., Pollenz R.S. (1999). Aryl hydrocarbon receptor imported into the nucleus following ligand binding is rapidly degraded via the cytosplasmic proteasome following nuclear export. J. Biol. Chem..

[33] Roberts B.J., Whitelaw M.L. (1999). Degradation of the basic helix-loop-helix/Per-ARNT-Sim homology domain dioxin receptor via the ubiquitin/proteasome pathway. J. Biol. Chem..

[34] Ma Q., Baldwin K.T. (2000). 2,3,7,8-Tetrachlorodibenzo-p-dioxin-induced degradation of aryl hydrocarbon receptor (AhR) by the ubiquitin-proteasome pathway. Role of the transcription activation and DNA binding of AhR. J. Biol. Chem..

[35] Arpiainen S., Raffalli-Mathieu F., Lang M.A., Pelkonen O., Hakkola J. (2005). Regulation of the Cyp2a5 gene involves an aryl hydrocarbon receptor-dependent pathway. Mol. Pharmacol..

[36] Deb S., Bandiera S.M. (2010). Characterization of a new cytochrome P450 enzyme, CYP2S1, in rats: Its regulation by aryl hydrocarbon receptor agonists. Toxicology.

[37] Kalthoff S., Ehmer U., Freiberg N., Manns M.P., Strassburg C.P. (2010). Interaction between oxidative stress sensor Nrf2 and xenobiotic-activated aryl hydrocarbon receptor in the regulation of the human phase II detoxifying UDP-glucuronosyltransferase 1A10. J. Biol. Chem..

[38] Li C.Y., Renaud H.J., Klaassen C.D., Cui J.Y. (2016). Age-Specific Regulation of Drug-Processing Genes in Mouse Liver by Ligands of Xenobiotic-Sensing Transcription Factors. Drug Metab. Dispos..

[39] Ma Q., Pohjanvirta R. (2012). Overview of AHR functional domains and the classical AHR signaling pathway: Induction of drug metabolizing enzymes. The AH Receptor in Biology and Toxicology.

[40] Yeager R.L., Reisman S.A., Aleksunes L.M., Klaassen C.D. (2009). Introducing the “TCDD-inducible AhR-Nrf2 gene battery”. Toxicol. Sci..

[41] Boverhof D.R., Burgoon L.D., Tashiro C., Sharratt B., Chittim B., Harkema J.R., Mendrick D.L., Zacharewski T.R. (2006). Comparative toxicogenomic analysis of the hepatotoxic effects of TCDD in Sprague Dawley rats and C57BL/6 mice. Toxicol. Sci..

[42] Boutros P.C., Yan R., Moffat I.D., Pohjanvirta R., Okey A.B. (2008). Transcriptomic responses to 2,3,7,8-tetrachlorodibenzo-p-dioxin (TCDD) in liver: Comparison of rat and mouse. BMC Genom..

[43] Franc M.A., Moffat I.D., Boutros P.C., Tuomisto J.T., Tuomisto J., Pohjanvirta R., Okey A.B. (2008). Patterns of dioxin-altered mRNA expression in livers of dioxin-sensitive versus dioxin-resistant rats. Arch. Toxicol..

[44] Tijet N., Boutros P.C., Moffat I.D., Okey A.B., Tuomisto J., Pohjanvirta R. (2006). Aryl hydrocarbon receptor regulates distinct dioxin-dependent and dioxin-independent gene batteries. Mol. Pharmacol..

[45] Riddick D.S., Lee C., Bhathena A., Timsit Y.E., Cheng P.Y., Morgan E.T., Prough R.A., Ripp S.L., Miller K.K., Jahan A. (2004). Transcriptional suppression of cytochrome P450 genes by endogenous and exogenous chemicals. Drug Metab. Dispos..

[46] Mimura J., Ema M., Sogawa K., Fujii-Kuriyama Y. (1999). Identification of a novel mechanism of regulation of Ah (dioxin) receptor function. Genes Dev..

[47] MacPherson L., Tamblyn L., Rajendra S., Bralha F., McPherson J.P., Matthews J. (2013). 2,3,7,8-Tetrachlorodibenzo-p-dioxin poly(ADP-ribose) polymerase (TiPARP, ARTD14) is a mono-ADP-ribosyltransferase and repressor of aryl hydrocarbon receptor transactivation. Nucleic Acids Res..

[48] MacPherson L., Ahmed S., Tamblyn L., Krutmann J., Forster I., Weighardt H., Matthews J. (2014). Aryl hydrocarbon receptor repressor and TiPARP (ARTD14) use similar, but also distinct mechanisms to repress aryl hydrocarbon receptor signaling. Int. J. Mol. Sci..

[49] Matsumura F. (2009). The significance of the nongenomic pathway in mediating inflammatory signaling of the dioxin-activated Ah receptor to cause toxic effects. Biochem. Pharmacol..

[50] Matsumura F., Pohjanvirta R. (2012). Nongenomic route of action of TCDD: Identity, characteristics, and toxicological significance. The AH Receptor in Biology and Toxicology.

[51] Patel R.D., Murray I.A., Flaveny C.A., Kusnadi A., Perdew G.H. (2009). Ah receptor represses acute-phase response gene expression without binding to its cognate response element. Lab. Investig..

[52] Li W., Vogel C.F., Wu D., Matsumura F. (2010). Non-genomic action of TCDD to induce inflammatory responses in HepG2 human hepatoma cells and in liver of C57BL/6J mice. Biol. Chem..

[53] Tsai M.J., Hsu Y.L., Wang T.N., Wu L.Y., Lien C.T., Hung C.H., Kuo P.L., Huang M.S. (2014). Aryl hydrocarbon receptor (AhR) agonists increase airway epithelial matrix metalloproteinase activity. J. Mol. Med..

[54] Tanos R., Patel R.D., Murray I.A., Smith P.B., Patterson A.D., Perdew G.H. (2012). Aryl hydrocarbon receptor regulates the cholesterol biosynthetic pathway in a dioxin response element-independent manner. Hepatology.

[55] Ohtake F., Baba A., Takada I., Okada M., Iwasaki K., Miki H., Takahashi S., Kouzmenko A., Nohara K., Chiba T. (2007). Dioxin receptor is a ligand-dependent E3 ubiquitin ligase. Nature.

[56] Ohtake F., Kato S., Pohjanvirta R. (2012). The E3 ubiquitin ligase activity of transcription factor AHR permits nongenomic regulation of biological pathways. The AH Receptor in Biology and Toxicology.

[57] Weiss C., Faust D., Durk H., Kolluri S.K., Pelzer A., Schneider S., Dietrich C., Oesch F., Gottlicher M. (2005). TCDD induces c-jun expression via a novel Ah (dioxin) receptor-mediated p38-MAPK-dependent pathway. Oncogene.

[58] de Oliveira S.K., Smolenski A. (2009). Phosphodiesterases link the aryl hydrocarbon receptor complex to cyclic nucleotide signaling. Biochem. Pharmacol..

[59] Jin M.H., Hong C.H., Lee H.Y., Kang H.J., Han S.W. (2008). Enhanced TGF-beta1 is involved in 2,3,7,8-tetrachlorodibenzo-p-dioxin (TCDD) induced oxidative stress in C57BL/6 mouse testis. Toxicol. Lett..

[60] Salisbury T.B., Tomblin J.K., Primerano D.A., Boskovic G., Fan J., Mehmi I., Fletcher J., Santanam N., Hurn E., Morris G.Z. (2014). Endogenous aryl hydrocarbon receptor promotes basal and inducible expression of tumor necrosis factor target genes in MCF-7 cancer cells. Biochem. Pharmacol..

[61] Azkargorta M., Fullaondo A., Laresgoiti U., Aloria K., Infante A., Arizmendi J.M., Zubiaga A.M. (2010). Differential proteomics analysis reveals a role for E2F2 in the regulation of the Ahr pathway in T lymphocytes. Mol. Cell. Proteom..

[62] Elferink C.J., Ge N.L., Levine A. (2001). Maximal aryl hydrocarbon receptor activity depends on an interaction with the retinoblastoma protein. Mol. Pharmacol..

[63] Watabe T. (2011). Roles of old players in the suppression of a new player: Networks for the transcriptional control of angiogenesis. J. Biochem..

[64] Pääjärvi G., Viluksela M., Pohjanvirta R., Stenius U., Högberg J. (2005). TCDD activates Mdm2 and attenuates the p53 response to DNA damaging agents. Carcinogenesis.

[65] Backlund M., Ingelman-Sundberg M. (2005). Regulation of aryl hydrocarbon receptor signal transduction by protein tyrosine kinases. Cell. Signal..

[66] Sutter C.H., Yin H., Li Y., Mammen J.S., Bodreddigari S., Stevens G., Cole J.A., Sutter T.R. (2009). EGF receptor signaling blocks aryl hydrocarbon receptor-mediated transcription and cell differentiation in human epidermal keratinocytes. Proc. Natl. Acad. Sci. USA.

[67] Xie G., Peng Z., Raufman J.P. (2012). Src-mediated aryl hydrocarbon and epidermal growth factor receptor cross talk stimulates colon cancer cell proliferation. Am. J. Physiol. Gastrointest. Liver Physiol..

[68] Barhoover M.A., Hall J.M., Greenlee W.F., Thomas R.S. (2010). Aryl hydrocarbon receptor regulates cell cycle progression in human breast cancer cells via a functional interaction with cyclin-dependent kinase 4. Mol. Pharmacol..

[69] Ke S., Rabson A.B., Germino J.F., Gallo M.A., Tian Y. (2001). Mechanism of suppression of cytochrome P-450 1A1 expression by tumor necrosis factor-alpha and lipopolysaccharide. J. Biol. Chem..

[70] Braeuning A., Kohle C., Buchmann A., Schwarz M. (2011). Coordinate regulation of cytochrome P450 1a1 expression in mouse liver by the aryl hydrocarbon receptor and the beta-catenin pathway. Toxicol. Sci..

[71] Hofer T., Pohjanvirta R., Spielmann P., Viluksela M., Buchmann D.P., Wenger R.H., Gassmann M. (2004). Simultaneous exposure of rats to dioxin and carbon monoxide reduces the xenobiotic but not the hypoxic response. Biol. Chem..

[72] Hwang J., Kleinhenz D.J., Rupnow H.L., Campbell A.G., Thule P.M., Sutliff R.L., Hart C.M. (2007). The PPARgamma ligand, rosiglitazone, reduces vascular oxidative stress and NADPH oxidase expression in diabetic mice. Vascul. Pharmacol..

[73] Shibahara N., Masunaga Y., Iwano S., Yamazaki H., Kiyotani K., Kamataki T. (2011). Human cytochrome P450 1A1 is a novel target gene of liver X receptor alpha. Drug Metab. Pharmacokinet..

[74] Brunnberg S., Swedenborg E., Gustafsson J.A., Pohjanvirta R. (2012). Functional interactions of AHR with other receptors. The AH Receptor in Biology and Toxicology.

[75] Tian Y., Ke S., Thomas T., Meeker R.J., Gallo M.A. (1998). Transcriptional suppression of estrogen receptor gene expression by 2,3,7,8-tetrachlorodibenzo-p-dioxin (TCDD). J. Steroid Biochem. Mol. Biol..

[76] Spink D.C., Hayes C.L., Young N.R., Christou M., Sutter T.R., Jefcoate C.R., Gierthy J.F. (1994). The effects of 2,3,7,8-tetrachlorodibenzo-p-dioxin on estrogen metabolism in MCF-7 breast cancer cells: Evidence for induction of a novel 17 beta-estradiol 4-hydroxylase. J. Steroid Biochem. Mol. Biol..

[77] Safe S., Wormke M., Samudio I. (2000). Mechanisms of inhibitory aryl hydrocarbon receptor-estrogen receptor crosstalk in human breast cancer cells. J. Mammary Gland Biol. Neoplasia.

[78] Matthews J., Wihlen B., Thomsen J., Gustafsson J.A. (2005). Aryl hydrocarbon receptor-mediated transcription: Ligand-dependent recruitment of estrogen receptor alpha to 2,3,7,8-tetrachlorodibenzo-p-dioxin-responsive promoters. Mol. Cell. Biol..

[79] Ruegg J., Swedenborg E., Wahlstrom D., Escande A., Balaguer P., Pettersson K., Pongratz I. (2008). The transcription factor aryl hydrocarbon receptor nuclear translocator functions as an estrogen receptor beta-selective coactivator, and its recruitment to alternative pathways mediates antiestrogenic effects of dioxin. Mol. Endocrinol..

[80] Kuo L.C., Cheng L.C., Lin C.J., Li L.A. (2013). Dioxin and estrogen signaling in lung adenocarcinoma cells with different aryl hydrocarbon receptor/estrogen receptor alpha phenotypes. Am. J. Respir. Cell Mol. Biol..

[81] Patrizi B., Siciliani de Cumis M. (2018). TCDD Toxicity Mediated by Epigenetic Mechanisms. Int. J. Mol. Sci..

[82] Garrison P.M., Denison M.S. (2000). Analysis of the murine AhR gene promoter. J. Biochem. Mol. Toxicol..

[83] Garrison P.M., Rogers J.M., Brackney W.R., Denison M.S. (2000). Effects of histone deacetylase inhibitors on the Ah receptor gene promoter. Arch. Biochem. Biophys..

[84] Beedanagari S.R., Taylor R.T., Bui P., Wang F., Nickerson D.W., Hankinson O. (2010). Role of epigenetic mechanisms in differential regulation of the dioxin-inducible human CYP1A1 and CYP1B1 genes. Mol. Pharmacol..

[85] Beedanagari S.R., Taylor R.T., Hankinson O. (2010). Differential regulation of the dioxin-induced Cyp1a1 and Cyp1b1 genes in mouse hepatoma and fibroblast cell lines. Toxicol. Lett..

[86] Ko C.I., Puga A., Pohjanvirta R. (2012). Epigenetic mechanisms in AHR function. The AH Receptor in Biology and Toxicology.

[87] Kurita H., Schnekenburger M., Ovesen J.L., Xia Y., Puga A. (2014). The Ah receptor recruits IKKalpha to its target binding motifs to phosphorylate serine-10 in histone H3 required for transcriptional activation. Toxicol. Sci..

[88] Shen E.S., Whitlock J.P. (1989). The potential role of DNA methylation in the response to 2,3,7,8-tetrachlorodibenzo-p-dioxin. J. Biol. Chem..

[89] Wu Q., Ohsako S., Ishimura R., Suzuki J.S., Tohyama C. (2004). Exposure of mouse preimplantation embryos to 2,3,7,8-tetrachlorodibenzo-p-dioxin (TCDD) alters the methylation status of imprinted genes H19 and Igf2. Biol. Reprod..

[90] Yuan X., Qiu L., Pu Y., Liu C., Zhang X., Wang C., Pu W., Fu Y. (2016). Histone acetylation is involved in TCDDinduced cleft palate formation in fetal mice. Mol. Med. Rep..

[91] Wang C., Yuan X.G., Liu C.P., Zhai S.N., Zhang D.W., Fu Y.X. (2017). Preliminary research on DNA methylation changes during murine palatogenesis induced by TCDD. J. Craniomaxillofac. Surg..

[92] Ray S.S., Swanson H.I. (2004). Dioxin-induced immortalization of normal human keratinocytes and silencing of p53 and p16INK4a. J. Biol. Chem..

[93] Singh N.P., Singh U.P., Singh B., Price R.L., Nagarkatti M., Nagarkatti P.S. (2011). Activation of aryl hydrocarbon receptor (AhR) leads to reciprocal epigenetic regulation of FoxP3 and IL-17 expression and amelioration of experimental colitis. PLoS ONE.

[94] Gao L., Yin J., Wu W. (2016). Long non-coding RNA H19-mediated mouse cleft palate induced by 2,3,7,8-tetrachlorodibenzo-p-dioxin. Exp. Ther. Med..

[95] Moffat I.D., Boutros P.C., Celius T., Linden J., Pohjanvirta R., Okey A.B. (2007). microRNAs in adult rodent liver are refractory to dioxin treatment. Toxicol. Sci..

[96] Singh N.P., Singh U.P., Guan H., Nagarkatti P., Nagarkatti M. (2012). Prenatal exposure to TCDD triggers significant modulation of microRNA expression profile in the thymus that affects consequent gene expression. PLoS ONE.

[97] Hanieh H., Alzahrani A. (2013). MicroRNA-132 suppresses autoimmune encephalomyelitis by inducing cholinergic anti-inflammation: A new Ahr-based exploration. Eur. J. Immunol..

[98] Mocarelli P., Gerthoux P.M., Ferrari E., Patterson D.G., Kieszak S.M., Brambilla P., Vincoli N., Signorini S., Tramacere P., Carreri V. (2000). Paternal concentrations of dioxin and sex ratio of offspring. Lancet.

[99] Ryan J.J., Amirova Z., Carrier G. (2002). Sex ratios of children of Russian pesticide producers exposed to dioxin. Environ. Health Perspect..

[100] ‘t Mannetje A., Eng A., Walls C., Dryson E., Kogevinas M., Brooks C., McLean D., Cheng S., Smith A.H., Pearce N. (2017). Sex ratio of the offspring of New Zealand phenoxy herbicide producers exposed to 2,3,7,8-tetrachlorodibenzo-p-dioxin. Occup. Environ. Med..

[101] Murray F.J., Smith F.A., Nitschke K.D., Humiston C.G., Kociba R.J., Schwetz B.A. (1979). Three-generation reproduction study of rats given 2,3,7,8-tetrachlorodibenzo-p-dioxin (TCDD) in the diet. Toxicol. Appl. Pharmacol..

[102] Ikeda M., Tamura M., Yamashita J., Suzuki C., Tomita T. (2005). Repeated in utero and lactational 2,3,7,8-tetrachlorodibenzo-p-dioxin exposure affects male gonads in offspring, leading to sex ratio changes in F2 progeny. Toxicol. Appl. Pharmacol..

[103] Ishihara K., Warita K., Tanida T., Sugawara T., Kitagawa H., Hoshi N. (2007). Does paternal exposure to 2,3,7,8-tetrachlorodibenzo-p-dioxin (TCDD) affect the sex ratio of offspring?. J. Vet. Med. Sci..

[104] Ishihara K., Ohsako S., Tasaka K., Harayama H., Miyake M., Warita K., Tanida T., Mitsuhashi T., Nanmori T., Tabuchi Y. (2010). When does the sex ratio of offspring of the paternal 2,3,7,8-tetrachlorodibenzo-p-dioxin (TCDD) exposure decrease: In the spermatozoa stage or at fertilization?. Reprod. Toxicol..

[105] Ding T., McConaha M., Boyd K.L., Osteen K.G., Bruner-Tran K.L. (2011). Developmental dioxin exposure of either parent is associated with an increased risk of preterm birth in adult mice. Reprod. Toxicol..

[106] You Y.A., Mohamed E.A., Rahman M.S., Kwon W.S., Song W.H., Ryu B.Y., Pang M.G. (2018). 2,3,7,8-Tetrachlorodibenzo-p-dioxin can alter the sex ratio of embryos with decreased viability of Y spermatozoa in mice. Reprod. Toxicol..

[107] Grech V. (2014). Secular trends in newborn sex ratios. Early Hum. Dev..

[108] Terrell M.L., Hartnett K.P., Marcus M. (2011). Can environmental or occupational hazards alter the sex ratio at birth? A systematic review. Emerg. Health. Threats J..

[109] Nieminen P., Lehtiniemi H., Huusko A., Vahakangas K., Rautio A. (2013). Polychlorinated biphenyls (PCBs) in relation to secondary sex ratio--a systematic review of published studies. Chemosphere.

[110] Rowlands J.C., Budinsky R.A., Aylward L.L., Faqi A.S., Carney E.W. (2006). Sex ratio of the offspring of Sprague-Dawley rats exposed to 2,3,7,8-tetrachlorodibenzo-p-dioxin (TCDD) in utero and lactationally in a three-generation study. Toxicol. Appl. Pharmacol..

[111] Manikkam M., Tracey R., Guerrero-Bosagna C., Skinner M.K. (2012). Dioxin (TCDD) induces epigenetic transgenerational inheritance of adult onset disease and sperm epimutations. PLoS ONE.

[112] Manikkam M., Guerrero-Bosagna C., Tracey R., Haque M.M., Skinner M.K. (2012). Transgenerational actions of environmental compounds on reproductive disease and identification of epigenetic biomarkers of ancestral exposures. PLoS ONE.

[113] Nilsson E., Larsen G., Manikkam M., Guerrero-Bosagna C., Savenkova M.I., Skinner M.K. (2012). Environmentally induced epigenetic transgenerational inheritance of ovarian disease. PLoS ONE.

[114] Bruner-Tran K.L., Osteen K.G. (2011). Developmental exposure to TCDD reduces fertility and negatively affects pregnancy outcomes across multiple generations. Reprod. Toxicol..

[115] Bruner-Tran K.L., Ding T., Yeoman K.B., Archibong A., Arosh J.A., Osteen K.G. (2014). Developmental exposure of mice to dioxin promotes transgenerational testicular inflammation and an increased risk of preterm birth in unexposed mating partners. PLoS ONE.

[116] Ding T., Mokshagundam S., Rinaudo P.F., Osteen K.G., Bruner-Tran K.L. (2018). Paternal developmental toxicant exposure is associated with epigenetic modulation of sperm and placental Pgr and Igf2 in a mouse model. Biol. Reprod..

[117] Sanabria M., Cucielo M.S., Guerra M.T., Dos Santos Borges C., Banzato T.P., Perobelli J.E., Leite G.A., Anselmo-Franci J.A., De Grava Kempinas W. (2016). Sperm quality and fertility in rats after prenatal exposure to low doses of TCDD: A three-generation study. Reprod. Toxicol..

[118] Olsvik P.A., Williams T.D., Tung H.S., Mirbahai L., Sanden M., Skjaerven K.H., Ellingsen S. (2014). Impacts of TCDD and MeHg on DNA methylation in zebrafish (Danio rerio) across two generations. Comp. Biochem. Physiol. C Toxicol. Pharmacol..

[119] Baker T.R., King-Heiden T.C., Peterson R.E., Heideman W. (2014). Dioxin induction of transgenerational inheritance of disease in zebrafish. Mol. Cell. Endocrinol..

[120] Meyer D.N., Baker B.B., Baker T.R. (2018). Ancestral TCDD Exposure Induces Multigenerational Histologic and Transcriptomic Alterations in Gonads of Male Zebrafish. Toxicol. Sci..

[121] Mendelson C.R. (2009). Minireview: Fetal-maternal hormonal signaling in pregnancy and labor. Mol. Endocrinol..

[122] Wang X., Miller D.C., Harman R., Antczak D.F., Clark A.G. (2013). Paternally expressed genes predominate in the placenta. Proc. Natl. Acad. Sci. USA.

[123] Bidgoli S.A., Karimi M., Asami Z., Baher H., Djamali Zavarhei M. (2011). Association between testicular Aryl hydrocarbon Receptor levels and idiopathic male infertility: A case-control study in Iran. Sci. Total Environ..

[124] Hansen D.A., Esakky P., Drury A., Lamb L., Moley K.H. (2014). The aryl hydrocarbon receptor is important for proper seminiferous tubule architecture and sperm development in mice. Biol. Reprod..

[125] Hurst C.H., DeVito M.J., Setzer W., Birnbaum L.S. (2000). Acute administration of 2,3,7,8-tetrachlorodibenzo-p-dioxin (TCDD) in pregnant Long-Evans rats: Association of measured tissue concentrations with developmental effects. Toxicol. Sci..

